# The influence of patient-physician communication on physician loyalty and hospital loyalty of the patient

**DOI:** 10.12669/pjms.344.15136

**Published:** 2018

**Authors:** Ozgun Unal, Mahmut Akbolat, Mustafa Amarat

**Affiliations:** 1 Ozgun Unal, PhD Student. Department of Health Care Management, Sakarya University, Sakarya, Turkey; 2 Dr. Mahmut Akbolat, Associate Professor, Department of Health Care Management, Sakarya University, Sakarya, Turkey; 3 Mustafa Amarat, PhD Student. Department of Health Care Management, Sakarya University, Sakarya, Turkey

**Keywords:** Loyalty to Hospital, Loyalty to Physician, Physician Patient Communication

## Abstract

**Objective:**

Patient-physician communication is important for an effective healthcare service and for the patient’s development of loyalty to the hospital. In this regard, this study aimed to determine whether there is a relationship between the patient-physician communication, the loyalty of the patient to the physician and to the hospital. Also study aimed to determine whether there is a mediating role of the physician loyalty on the patient-physician communication effect on to the hospital loyalty.

**Method:**

Five hundred ten questionnaires were distributed to regular public patients of the government hospitals, clinics, and private clinic patients in Sakarya using a simple random sampling method. Data were analysed using descriptive statistics and Structural Equation Modelling (SEM).

**Results:**

According to the findings, there was a significant relationship between patient-physician communication and loyalty to physician and to hospital. Patient-physician communication has a significant effect on loyalty to physician and hospital. In addition, patient loyalty has a mediating role on the patient-physician communication effect on the hospital loyalty.

**Conclusion:**

According to the result of the study, physician-patient communication could be used as an important tool in creating physician loyalty and hospital loyalty. This study helps physicians and health service providers to formulate strategies and tactics that will effectively develop the loyalty of patients.

## INTRODUCTION

Patient-physician communication (PPC) is the type of communication established between physicians who provide health service and determine the quality as well as the content of the provided service, and the health service users. To be able to bring physicians benefit to their patients at the highest level, they not only need their technical knowledge but also effective communication skill.^1^

Effective communication can increase patient participation in treatment and satisfaction with the service they receive and this can positively impact treatment outcomes.^2^,^3^ Effective patient-physician communication leads individuals to feel comfortable, participate in treatment, have a more successful and positive treatment process, have confidence in the service provider with positive treatment results, and satisfaction of them with the service they have received.^4^,^5^ On the contrary the inability to establish an effective communication between the patient and the physician causes the physician to be unable to provide the patient with sufficient benefits and results in the dissatisfaction of the patient.^6^-^8^ Studies show that satisfied patients are more inclined to pay for services and products.^9^

Loyalty is the behaviour of the customer to continue receiving service from a service provider.^10^ Loyalty to health services can be defined as tendency of re-selection of same institution to meet future healthcare needs by individuals due to their satisfaction from past experience as well as their trust in service provider and healthcare professionals. Satisfaction is also an important sign of patient loyalty.^11^,^12^ Two types of loyalty can be mentioned in the healthcare provision. The first is loyalty to the physician (PL) and it can be described that the patient is satisfied with the service of the physician and continues to receive services from that physician in prospective similar health needs. The other is loyalty to the hospital (HL) and it may be influenced due to PL or other factors which include service quality, service diversity, modern physical facilities, the level of development of equipment, courtesy, dedication of employees, and additional fees to be paid.^13^

Since one of key factors of healthcare institutions is to have loyal healthcare users to be able to survive against their competitors, this study was designed to examine the effect PPC on PL and HL.

This study looked at more comprehensive model of the simultaneous effects of several key antecedents by examining the integrative system of the relationships. Furthermore, the study incorporates communication as input into the model, thus this improves and generalizes findings as well as extending the theoretical base of health care research.

### Hypotheses

1. H_1_: There is a relationship between patient-physician communication and patient’s loyalty to physician.
2. H_2_: Patient communication affects the patient’s loyalty to the physician.
3. H_3_: There is a relationship between patient-physician communication and the patient’s loyalty to the hospital.
4. H_4_: Patient-physician communication affects the patient’s loyalty to the hospital.
5. H_5_: There is a relationship between the patient’s loyalty to the physician and the patient’s loyalty to the hospital.
6. H_6_: The patient’s loyalty to the physician affects the patient’s loyalty to the hospital.
7. H_7_: Physician loyalty mediates the effects of patient-physician communication on to loyalty to the hospital.

### Research Model

**Fig.1:**
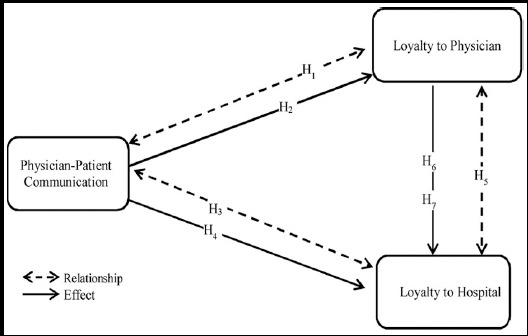
Research Model.

The following model was developed in the study after review of literature and developed hypotheses. (See Fig.1)

## METHODS

Before starting the study was approved by the Ethics Committee of Sakarya University and it in compliance with the ethical principles (EC approval No: 61923333/050.99/54). The study was conducted between May 10, 2016 to July 10, 2016. Five hundred and ten questionnaires were distributed to regular public patients of the government hospitals, clinics, and private clinic patients in Sakarya using a simple random sampling method.

The reason why these patients were chosen is that they possessed the information required for the research project. All respondents are outpatients of government hospitals and clinics, or private clinics, qualifying them as these respondents have sufficient experience and knowledge to evaluate the service provided by their physicians. They were required to complete four sections of a questionnaire: demographic profile, Loyalty to Hospital Scale, Loyalty to Physician Scale and Patient Physician Communication scale. Respondents answered by agreeing or disagreeing with the statement using a Likert scale from 1=strongly disagree to 5=strongly agree. Data were analysed using descriptive statistics and Structural Equation Modelling (SEM).

## RESULTS

### Validity and Reliability

In this study, Cronbach Alpha value was used, and The Cronbach Alpha value for the HL Scale, the PL Scale, and the PPC Scale were 0.947, 0.872, and 0.882, respectively. According to these findings, the scale has the necessary conditions for reliability. The results obtained from the study indicates that the data set is suitable for factor analysis.

In the result of exploratory factor analysis of the scale, it is gathered under three dimensions to be able to explain PPC, PL and HL. The KMO value of the scale is 0.933, Bartlett’s Test of Sphericity is significant. The total variance explained in the scale is 59,034%. It seems that the scales meet all requirements.

After this step, confirmatory factor analysis was performed. Table-I illustrates, according to the literature, the lowest and highest values of the scales related to some goodness of fit^14^and the goodness of fit indexes obtained from this study.As the table suggests, goodness of fit indicates that the scale is at a usable level.

**Table-I:** Goodness of fit indexes.

Criteria of Fit	Acceptable values of fit	Goodness of fit
CMIN (p)	>0.05	691.59 (0.00)
DF (Degrees of freedom)	N.A	307
CMIN/DF	>3.0	2.25
GFI (Goodness-of-fit index)	0.90≤GFI≤0.95	0.91
NFI (Normed fit index)	0.90≤NFI≤0.95	0.92
TLI (Tucker-Levis index)	0.95≤NNFI≤0.97	0.95
CFI (Comparative fit index)	0.95≤CFI≤0.97	0.95
AGFI (Adjusted goodness of fit index)	0.85≤AGFI≤0.90	0.89
RMSEA (Root mean square error of approximation)	0.05≤RMSEA≤0.10	0.05

Source^14^-^17^

### Hypothesis testing

The conceptual model has been made feasible as path analysis. To estimate the model, maximum likelihood estimation method was used in AMOS (version 22.0) software. In addition, the maximum likelihood method assumes the multivariate normality of the data.^16^Also, the multivariate kurtosis of the data was found 296,957, and this suggests that data violate the multivariate normality a little. In order to resolve this problem, preliminary loading was performed using asymptotically distributed-free.^16^

However, Path analysis goodness of fit can be assessed by using multiple indexes. In this study, the model was evaluated using 12 indexes which are widely used, and these indexes are; CMIN (715,06), DF (308), p (0,00), CMIN/DF (2,32), GFI (0,90), AGFI (0,88), NFI (0,92), RFI(0,90), IFI (0,95), TLI (0,94), CFI (0,95) and RMSEA (0,05).^16^,^18^ Overall, the values above are consistent with the recommended cut-off values,^16^ and accordingly the data forming the model can be termed appropriate for the usable level.

The relationship between dimensions that form the model was examined after the feasibility of the model was seen in the study. There is a statistically significant relationship between all three sub scales forming the scale. Hypotheses H_1_, H_3_ and H_5_ were accepted from this finding.

**Fig.2:**
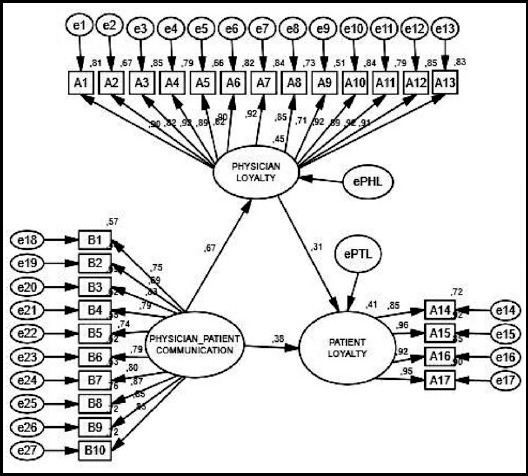
AMOS Output of the Model Test Showing the Influence of PPC on PL and HL

In the study, the path analysis model showing the direct effect of PPC on the PL and the indirect effect with mediating role of PL, as shown in Fig.2. According to path analysis results, there is statistically significant effect of PPC on PL (ρ = 0.670; t = 20.978) and HL (ρ = 0.383, t = 9.456). In addition, PL has a mediating role on PPC effect on HL (ρ = 0.314; t = 8.308). However, the mediator role of PL leads to a partial reduction in the level of influence of PPC, and therefore H2, H4, H6 and H7hypotheses were accepted.

## DISCUSSION

The most important factor that builds customer loyalty in service enterprises is employees who provide services.^19^ Loyalty develops as patients visit a particular healthcare professional (HCP) more often than others and become committed to that HCP.^20^ Patient-physician communication affects patient satisfaction, trust, treatment participation, efficacy of treatment, successful treatment outcomes and PL.^21^,^22^ A study conducted in Turkey has revealed that physician and patient communication is an important factor in PL.^23^ The results of current study has confirmed this knowledge. There is a significant relationship between PPC, PL and HL. In addition, PPC affects HL and PL positively. In this respect, similar results were found in the literature.^23^-^26^

Another result of the study showed that PL affects HL positively. This explains that there is a role of employees in customer loyalty and the finding is in line with past studies which were conducted in different areas.^19^, ^27^ In addition, PL has a mediating role on PPC effect on HL, so this is remarkable in that it shows how important patient communication is regarding hospitals because patients follow physicians. In other words, they prefer the hospital where their physician works. Thus, it can be said that it brings advantage in competitive industry for hospitals. Also, if hospitals cannot keep physicians who have loyal patients on their list or if they lose them to their opponents, it can also result in a disadvantageous situation.

## CONCLUSION

The results of the study demonstrate the importance of the patient-physician communication regarding health institutions. As such it is important that the health institutions that carry out their activities with the goal of profitability and continuity can achieve these goals not only by improving the quality of the offered service, but also by improving patient communication. It is also significant that physicians are the most important actors in communication with the patient. For this reason, it is necessary to take the communication skills along with the occupational skills of physicians into consideration. In this context, it can be suggested that physicians should be educated on communication skills during their formal education as well as in-service trainings, and also trained in communicating with patients. In addition, it is possible to make patients and patient relatives comply with the advice and treatment faster by educating them on communication with the physicians and other health professionals in health institutions that have developed in recent years.

### Author`s Contribution

**OU, MAK** conceived, designed and did statistical analysis & editing of manuscript.

**OU, MAK** did data collection and manuscript writing.

**MAM** did review and final approval of manuscript.
